# Isolation of *Klebsiella pneumoniae* Phage vB_KpnS_MK54 and Pathological Assessment of Endolysin in the Treatment of Pneumonia Mice Model

**DOI:** 10.3389/fmicb.2022.854908

**Published:** 2022-03-21

**Authors:** Biao Lu, Xueping Yao, Guangli Han, Zidan Luo, Jieru Zhang, Kang Yong, Yin Wang, Yan Luo, Zexiao Yang, Meishen Ren, Suizhong Cao

**Affiliations:** ^1^College of Veterinary Medicine, Sichuan Agricultural University, Chengdu, China; ^2^Key Laboratory of Animal Disease and Human Health of Sichuan Province, Chengdu, China; ^3^College of Animal Science and Technology, Chongqing Three Gorges Vocational College, Chongqing, China

**Keywords:** *Klebsiella pneumoniae*, phage, endolysin, *in vitro* tests, *in vivo* tests, antibacterial activity

## Abstract

With the improper use of antibiotics, an increasing number of multidrug-resistant bacteria have been reported worldwide, posing challenges for disease treatment. *Klebsiella pneumoniae* is an important zoonotic pathogen that colonises the respiratory tract. Endolysin therapy has emerged with the development of phages. In this study, a lytic phage vB_KpnS_MK54 was isolated from the drinking water of a forest musk deer (FMD) farm in Sichuan Province. It was the first reported phage obtained from FMD. The primary biological characteristics were determined, and whole-genome sequencing analysis was performed. The phage which belongs to the family Siphoviridae is highly specific for lytic host bacteria and is moderately adaptable to different environments. Whole-genome sequencing results showed that the phage genome size was 46,218 bp. There were 80 coding DNA sequences (CDSs) in total, 32 of which had known functions. The last CDS is the phage endolysin LysG24. A new peptide-modified endolysin (LysCA) was constituted by connecting the cecropin A peptide residues with LysG24 to investigate the antibacterial activities of both LysG24 and LysCA. The results showed that the lytic profile of LysG24 and LysCA was wider than that of phage MK54. For *in vitro* tests, both endolysins destroyed 99% of the host bacteria within 6 h. The lysing ability and environmental adaptability of LysCA were significantly stronger than those of LysG24. For *in vivo* tests, LysG24 and LysCA exhibited therapeutic effects in a mouse model of pneumonia wherewith the mice were infected with *K. pneumoniae* (LPKP), wherein both LysG24 and LysCA can effectively reduce the pulmonary inflammatory response. The LPKP bacterial load in the treatment group was significantly lower than that in the bacterial group, among which LysCA displayed a more obvious therapeutic effect. Furthermore, the safety test showed that the endolysins had no toxic effects on mice. In general, both LysG24 and LysCA showed excellent antibacterial activity *in vivo* and *in vitro*, with high safety and strong adaptability to the environment, manifesting their latent potential as new antimicrobial agents.

## Introduction

The forest musk deer (*Moschus berezovskii*) is a vital protected animal in China farmed on a large scale because of its economic value. However, respiratory diseases have restricted the development of FMD captive breeding. *K. pneumoniae* is an important pathogen which causes respiratory sickness in FMD (Zhao et al., 2021). *K. pneumoniae* belongs to the genus *Klebsiella Trevisan* in the Enterobacteriaceae family, and it is an essentially conditional pathogen that exists widely in nature (Zhao et al., 2017). *K. pneumoniae* can cause infection when the immune system of humans or animals is weakened or the florae are imbalanced in the body, causing several diseases and even death in humans and animals with high morbidity and mortality (Brisse and Duijkeren, 2005; Yang et al., 2021). Antibiotics are the first option for the cure of bacterial pneumonia. However, improper use of antibiotics has led to widespread *K. pneumonia* resistance. The emergence of extension-spectrum β-lactamase, combined with the emergence of carbapenem-resistant bacteria, will make the treatment and control of *K. pneumoniae* infection significantly more difficult (Pires et al., 2020). To avoid the emergence of super-resistant bacteria and respond to the global call for resistance reduction, there is an urgent need to find and develop novel and effective antibacterial compounds. As such, phages may play an essential role in responding to this crisis.

Bacteriophages (phages) are viruses that exist naturally and specifically infect host bacteria. According to the mechanism by which they infect bacteria, they can be divided into lysogenic bacteriophages and lytic bacteriophages. Lytic phages can substitute for antibiotics in the clinical treatment of bacterial diseases because of their direct entry into the host bacteria for replication and lysis (Kakasis and Panitsa, 2019; Pires et al., 2020). Endolysin is a key element of the bacteriophage lysis system. It is a lytic enzyme produced at the later lysis stage after the phage infects the bacteria and assists in the release of progeny phages by degrading the peptidoglycan present in the bacteria (Young, 2014; Li et al., 2021). In addition, endolysin has high safety characteristics, low molecular weight and strong bactericidal ability (Dufour and Debarbieux, 2017). Recent studies have shown that natural endolysin is an effective antibacterial drug for the therapy of Gram-positive pathogen infections (Pastagia et al., 2013; Bae et al., 2019; Imanishi et al., 2019). However, the barrier formed by the outer membrane of Gram-negative bacteria can hinder the approach of endolysin, weakening its therapeutic effect. Several methods have been employed to help natural endolysin disorganise the barrier of the outer membrane of Gram-negative bacteria. First, the outer membrane penetrant improves the diffusion of endolysin. Natural endolysin can strengthen its ability to lyse host bacteria in an organic acid (5 mm malic acid, 2 mm citric acid or 0.5 mm EDTA) environment (Oliveira et al., 2014). Another method is to facilitate the penetration of endolysin through the outer membrane by designing proteins including antimicrobial peptides, such as SMAP-29 (Briers et al., 2014a) and cecropin A (Yang et al., 2015), that destabilise the outer membrane to modify the natural endolysin to break the outer membrane barrier and effectively kill Gram-negative bacteria. Therefore, methods to prevent and control endolysins in phages based on drug-resistant bacteria are also attracting attention.

In this study, *K. pneumoniae* was used as an indicator bacterium to isolate the lytic phage vB_KpnS_MK54 from an FMD farm in Sichuan Province. This phage has strong specificity and a good bacteriostasis effect, enabling the use of its lysin to prevent harm to FMD from *K. pneumoniae* as a starting point for subsequent research. Although many studies have examined peptide-modified lysins, most are limited to *in vitro* studies (Yang et al., 2015; Son et al., 2021). In the current study, endolysins were studied *in vivo*, with the aim of providing a reference point for subsequent endolysin-related studies. The main biological characteristics of the phage were detected, and its whole genome was sequenced. LysG24 was identified based on the whole-genome sequence and fused with cecropin A residues 1–8 (KWKLFKKI) to form a new peptide-modified lysin, LysCA. The bacteriostatic activities of LysG24 and LysCA were detected both *in vitro* and *in vivo*.

## Materials and Methods

### Bacterial Strains and Sample Processing

The bacterial strain used in this study was maintained at the Animal Quarantine Laboratory of Sichuan Agricultural University. Before using these strains, they were identified by bacterial 16S rRNA polymerase chain reaction (PCR) analysis (Ibal et al., 2019). The primers used are shown in Table 1. SM buffer, Luria-Bertani (LB) broth medium and agar powder used in the experiments were all from Solarbio Life Sciences (Beijing, China).

**Table 1:** Sequences of primers used in the experiment.

Name	Primer sequences (5'-3')	Fragment size
16S rRNA	F: AGAGTTTGATCCTGGCTCAG R: GGTTACCTTGTTACGACTT	1,465 bp
LysG24	F:CCGGAATTCATGAAACTATCAACGCG R:CCCAAGCTTTCATGACATGTATACCTC	456 bp
LysCA	F:CCGGGAATTCAAATGGAAATTATTTAAGAAAATTATGAAACTATCAACGC R:CCCAAGCTTTCATGACATGTATACCTCACG	481 bp
T7	F: TAATACGACTCACTATAGGG R: GCTACTTATTGCTCAGCGG	712 bp

LB soft agar (0.6% w/v): Add 0.6 g of agar powder to 100 ml of LB broth medium and autoclave at 121°C for 20 min.

LB agar plates (1.2% w/v): Add 1.2 g of agar powder to 100 ml of LB broth medium and autoclave at 121°C for 20 min.

### Isolation and Main Biological Characteristics

The separation and purification of bacteriophages were carried out using the double-layer plate method (Karumidze et al., 2013). The collected water samples (from a FMD farm in Sichuan) were mixed with the indicator bacterium *K. pneumoniae* (LPKP) and shaken for 4 h. The mixture was then filtered to obtain the phage stock solution. First, the phage stock solution and LPKP were mixed in equal proportions and incubated at 37°C for 20 min, followed by the addition of a 10-fold volume of 65°C fresh LB soft agar. Then, the mixture was immediately poured into LB agar plates to form a double-layer plate and incubated at 37°C for 10 h to observe the presence of plaques. For the purification of phages, single phage spots in the upper medium were picked and incubated in 1 ml of SM buffer at 4°C overnight. Subsequently, the phages were fully dissolved in the buffer and centrifuged at 8000 rpm for 5 min. The supernatant was diluted and purified using the double-layer plate method. To obtain the pure phage, the purification procedure was repeated five to six times until a phage spot of uniform shape and size was observed.

Phage concentration using polyethylene glycol (PEG) precipitation particle method. The purified phages were dropped into the carbon-coated copper mesh and placed for 10 min. The excess phage suspension was removed from the copper mesh with filter paper and dried for 1 min. Add 1 drop of 2% phosphotungstic acid solution (PTA, pH = 7.0) for staining, dried naturally, and phages were observed using a transmission electron microscope (H-7100FA, Hitachi, China). The bacteria used for the phage lysis profile assay included five strains of *K. pneumoniae*, three strains of *Escherichia coli*, three strains of *Staphylococcus aureus* and one strain each of *Bordetella bronchiseptica*, *Pseudomonas aeruginosa* and *Klebsiella oxytoca*. The double-layer plate method was used to determine whether the phage MK54 would lyse the test bacteria. The presence of plaque proves the lytic effect of phages on bacteria. Phage suspensions (8 × 10^13^ PFU/ml) were incubated at 40°C, 50°C, 60°C, 70°C and 80°C. Samples for each temperature were taken at 20, 40 and 60 min. The phages were then incubated in suspension buffers at pH 3, 5, 7, 9 and 11 for 1 h. The residual phage titre was verified using the double-layer plate count.

The multiplicity of infection (MOI) is the ratio of the number of phages to host bacteria when the phage infects the host bacteria. After the phage and LPKP was mixed at MOIs of 0.01, 0.1, 1, 10 and 100, the mixtures were incubated at 37°C for 8 h and then centrifuged at 8000 rpm for 5 min. This supernatant was filtered through a 0.22 μm Millipore filter, and the phage titre was detected to determine the best MOI. The optimal MOI could maximise the lytic efficiency of the phage.

The LPKP, in the log phase, and the phages (10^10^ PFU/ml) were mixed according to the optimal MOI. The mixture was then incubated at 37°C. The resultant solutions were collected at 10, 20, 40, 60, 90, 120 and 180 min. After 0.22 μm filtration, the phage titre was determined to draw the one-step growth curve of the phage. The calculation formula of lysis amount: lysis amount = phage titre at the end of lysis/concentration of host bacteria at the initial stage of infection.

### Phage Whole-Genome Sequencing Analysis

Phage DNA was extracted and purified from phage lysates using the Viral RNA/DNA Extraction Kit (Ver.5.0, TaKaRa MiniBEST), and the genome was verified by 1% agarose gel electrophoresis and sent to Shanghai Personal Biotechnology Co., Ltd. (Shanghai, China) for sequencing, and the results were analysed using GeneMarkS^1^ and DIAMOND^2^ to predict and annotate the protein-coding genes of the phage genome. The phage whole-genome sequence was uploaded to National Center for Biotechnology Information (NCBI) to obtain the serial number. According to the similarity of the genome sequence of MK54 to known sequences, then, create the map with genomic comparisons between analysed phage and reference phages selected from NCBI database as the most similar. We used CG View^3^ (Grant and Stothard, 2008) to construct the phage whole-genome map. The amino acid sequence of the main capsid protein and the large terminase subunit were used to construct a genetic phylogenetic tree using Molecular Evolutionary Genetics Analysis Version 7.0 (MEGA 7; Kumar et al., 2016).

### Construction of Plasmids and Protein Purification

Cecropin A is a 37-residue membrane-active antimicrobial polypeptide that kills bacteria by dissipating transmembrane electrochemical ion-gradients (Yang et al., 2015). Because the N-terminal residue of cecropin A (CA, KWKLFKKI) is highly positively charged, it binds to the negatively charged membrane lipid to form a dense layer of peptide, rendering it permeable. The base of cecropin A peptide was designed on the primer, and it was connected to the target fragment LysG24 by PCR reaction to form LysCA. Then, two target sequences and pET-32a (+) plasmid were double digested with *Hind* III and *EcoR* I restriction enzymes. Under the action of DNA ligase, the target fragment is ligated to the plasmid vector through cohesive end complementation. The primers used are listed in Table 1. After verification by sequencing, the correct plasmid was cloned and transformed into BL21 competent cells for protein expression.

The recombinant endolysin LysG24 and LysCA expression conditions were optimised for protein expression and purification in the laboratory. Firstly, 1 mm isopropyl β-D-thiogalactoside was added to the recombinant bacteria in the logarithmic phase to induce expression for 6 h. The expression product was then lysed by ultrasonic disruption. The recombinant protein was purified using a Ni-NTA gravity column, and an eluent containing 100 mm imidazole was used. The target protein was pooled and centrifuged in a 15 ml/10 kd Millipore ultrafiltration centrifuge tube at 3500 × g and 4°C for 30 min. The purified fragments were verified using SDS-PAGE.

### *In vitro* Antibacterial Activity

The *in vitro* antibacterial activity of recombinant endolysin was detected using the plate counting method and inhibition zone method. Since Gram-negative bacteria have outer membranes, we added 0.5 mm EDTA as outer membrane penetrants during the experiment. Plate counting method: The LPKP solution that grew in the logarithmic phase was taken as the initial data. The LPKP, recombinant endolysin (100 μg/ml) and 0.5 mmol/l EDTA were mixed in a ratio of 2:1:1 as the test group; PBS was used as a negative control and lysozyme was used as a positive control. The samples were taken at 0.5, 1, 2, 3, 4 and 6 h for counting to observe the changes in the number of viable LPKP. Inhibition zone method: 400 μl LPKP of log phase was added to 40 ml LB soft agar that was heated to liquid and poured the mixture on a plate after mixing well. A sterile Oxford cup was then inserted vertically into the LB medium. After the agar was completely solidified, the Oxford cup was gently pulled out, followed by the addition of 10 μl recombinant protein (100 μg/ml) and 10 μl EDTA to the small holes. The control groups were treated with lysozyme or PBS. Antimicrobial activity was evaluated by measuring the antimicrobial zone.

The optimal temperature, optimal pH value and lysis profile of the recombinant endolysin were determined using the plate counting method. The test bacteria were the same as the test bacteria for phage lysis profile. The recombinant endolysin and the test bacteria were mixed and cultured for 4 h, and the number of viable bacteria before and after mixing was calculated. A colony reduction of more than 50% was considered to have a lytic effect on the bacterium. We took 5 tubes of 1 ml logarithmic phase LPKP, and after changing the liquid, resuspended them in PBS buffer with pH values of 3, 5, 7, 9 and 11. Subsequently, 500 μl recombinant endolysins and 500 μl EDTA were added to the processed LPKP solution. In addition, the recombinant proteins were treated at 30°C, 37°C, 45°C, 55°C and 70°C for 1 h. Then, 500 μl of the treated recombinant endolysins and 500 μl of EDTA were mixed and then added to 1 ml LPKP. This was then incubated at 37°C for 4 h, and LPKP concentration was identified before and after mixing. The antibacterial activity of the recombinant endolysins was then calculated.

The endolysin antibacterial activity was equal to the ratio of the decrease in LPKP concentration of each test group to the initial LPKP concentration in the test.

### *In vivo* Antibacterial Activity

LPKP was used to construct a mouse pneumonia model through intranasal infection, and the therapeutic effects of LysG24 and LysCA on pneumonia were observed (Anand et al., 2020). BALB/c mice of either sex (weight 18–22 g) were used in this study. The animal handling procedures, including anaesthesia and sacrificing, were in accordance with the Animal Protection Law of the People’s Republic of China and approved by the National Institute of Animal Health Animal Care and Use Committee at Sichuan Agricultural University (no. SYXK2019-187). Thirty-six mice were randomly divided into six groups (six mice per group). Group A corresponded to the controls, while group B corresponded to bacteria. Groups C and D correspond to LysCA and LysG24 (Safe), respectively. Group E corresponded to bacteria+LysCA (Treat) while group F corresponded to bacteria + LysG24 (Treat). The intranasal infection dose of mice was 20 μl, and the concentration of LPKP was 10^9^ CFU/ml (Zhao et al., 2017). The therapeutic dose for intranasal delivery was 10 μl LysG24/LysCA (100 μg/ml) + 10 μl EDTA (0.5 mmol/L). The mice were reared for 48 h in the same environment to observe their clinical symptoms, and the mice were anaesthetised using 10% chloral hydrate (0.5 ml/100 g) and sacrificed.

The treatment effect was analysed based on the changes in the bacterial load and pathological changes in the lung tissues. The lungs of the mice were aseptically collected, weighed and ground into a tissue homogenate in an equal amount of sterile PBS. The tissue homogenate was diluted, and plate counting was performed. In addition, lung tissues with obvious lesions were selected and placed in 4% paraformaldehyde. Tissue sections were processed and analysed pathologically. The pathological sections were scored using Ishak standards.

### Statistical Analysis

All assays were performed at least three times in biological repeats. The experimental results were statistically analysed using GraphPad Prism (version 7.0, GraphPad Software, La Jolla, California), and the experimental data were analysed by one-way analysis of variance to compare significant differences. Statistical significance was set at *p* < 0.05.

## Results

### Phage Isolation and Main Biological Characteristics

#### Morphology of Phage vB_KpnS_MK54

**Figure 1:**
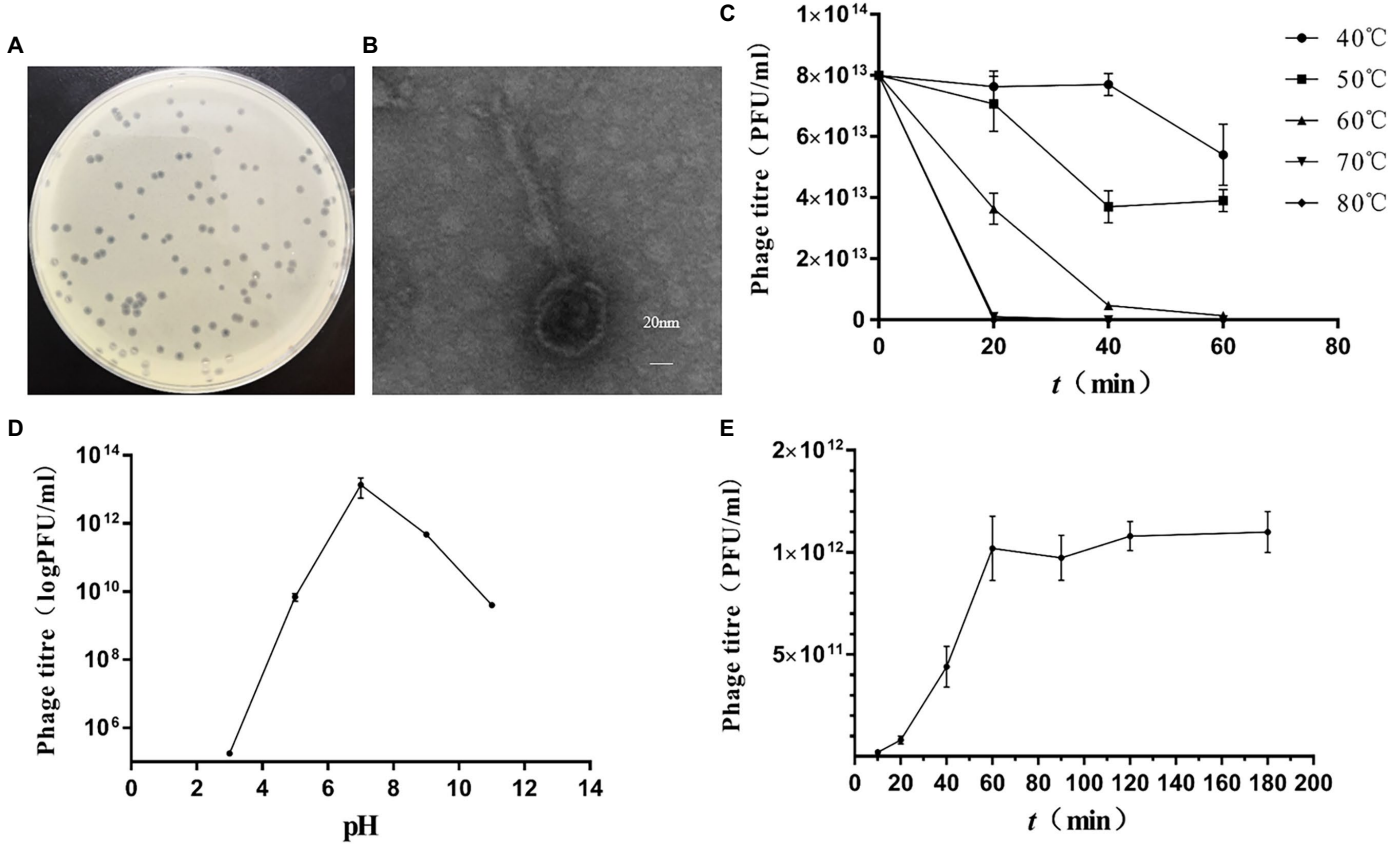
Isolation of *Klebsiella pneumoniae* phage vB_KpnS_MK54. **(A)** Plaques of phage. **(B)** Transmission electron micrograph of phage. **(C)** Phage activity at different temperatures. **(D)** Phage activity at different pH. **(E)** One-step growth curve of phage.

A lysate phage, vB_KpnS_MK54, was isolated from the drinking water of a FMD farm in Sichuan Province using *K. pneumoniae* LPKP as an indicator bacterium. It was the first reported phage obtained from FMD. A transparent plaque with a smooth edge, consistent size and 1-mm diameter was obtained after repeated purification (Figure 1A) with shallow halo rings. As observed using a transmission electron microscope, the head diameter of vB_KpnS_MK54 was approximately 60 nm, the tail length was 160 nm and the width was 10 nm (Figure 1B). According to the International Virus Classification Method developed by ICTV (Lefkowitz et al., 2018), vB_KpnS_MK54 belongs to the family Siphoviridae.

#### Lysis Profile and Stability

The lysis profile assay results showed that the phage only lysed the indicator bacterium LPKP and could not lyse other tested bacteria (Table 2). This indicates that vB_KpnS_MK54 has strong host specificity.

**Table 2:** Host range of phage vB_KpnS_MK54.

Number	Bacteria	Source	vB_KpnS_MK54
LPKP	*Klebsiella pneumoniae*	Forest musk deer	+
KP 1	*Klebsiella pneumoniae*	cattle	−
KP 2	*Klebsiella pneumoniae*	cattle	−
KP 3	*Klebsiella pneumoniae*	cattle	−
KP 4	*Klebsiella pneumoniae*	cattle	−
KP 5	*Klebsiella pneumoniae*	pig	−
PA 1	*Pseudomonas aeruginosa*	Forest musk deer	−
Bb 1	*Bordetella bronchiseptica*	Forest musk deer	−
KO 1	*Klebsiella oxytoca*	Forest musk deer	−
E.coli 1	*Escherichia coli*	Forest musk deer	−
E.coli 2	*Escherichia coli*	Forest musk deer	−
E.coli 3	*Escherichia coli*	cattle	−
SA 1	*Staphylococcus aureus*	Forest musk deer	−
SA 2	*Staphylococcus aureus*	Forest musk deer	−
SA 3	*Staphylococcus aureus*	cattle	−

+ Injection.

− Without injection.

Phage plaques were counted at different temperatures and times (Figure 1C). The figure shows that the phage survives in an environment with a relatively wide temperature range, but its titre will continue to decrease as the temperature rises. When the ambient temperature reaches 60°C, the phage titre drops significantly; when the environment temperature reaches 70°C and above, MK54 can be completely inactivated within 20 min. Phage plaques were counted at different pH values (Figure 1D). The results show that MK54 survives in a pH 5.0–11.0 environment and has a strong lysis ability. At pH < 5, bacteriophage MK54 loses its lysis ability.

#### MOI and One-Step Growth Curve

The optimal multiplicity of infection results showed that vB_KpnS_MK54 had the highest titre at MOI = 0.01, which is the optimal multiplicity of phage infection (Table 3).

**Table 3:** Determination of MOI of phage vB_KpnS_MK54.

MOI	Bacteria/CFU	Phage/PFU	Phage titre/(PFU/ml)
0.01	2 × 10^10^	2 × 10^8^	7.2 × 10^18^
0.1	2 × 10^10^	2 × 10^9^	2.3 × 10^17^
1	2 × 10^10^	2 × 10^10^	1.3 × 10^18^
10	2 × 10^10^	2 × 10^11^	1.7 × 10^17^
100	2 × 10^10^	2 × 10^12^	2.7 × 10^17^

MOI, multiplicity of infection.

According to the one-step growth curve (Figure 1E), the incubation period of phage MK54 was 20 min, the lysis period was 20–60 min and the lysis amount was 60 PFU/cell.

### Genome Analysis of vB_KpnS_MK54

**Figure 2:**
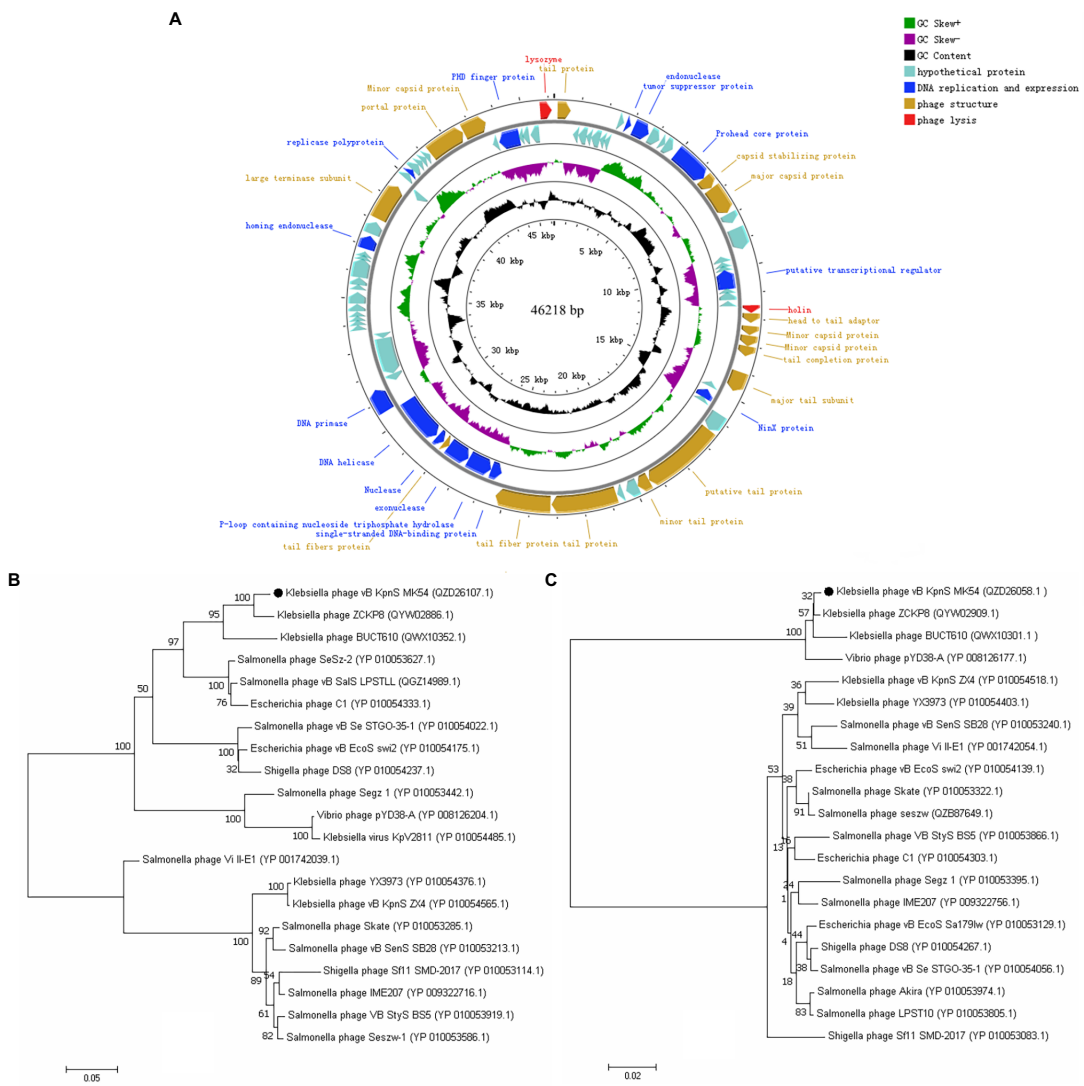
Complete genome and phylogenetics trees of phage vB_KpnS_MK54. **(A)** The outermost circle is the 80 CDS coded by vB_KpnS_MK54. The arrows represent the direction of gene transcription, and the colours represent genes with different functions: lysis-related genes (red), DNA replication and expression genes (blue), functional structural genes (brown) and hypothetical protein genes (cyan). Phylogenetics trees were formed based on the **(B)** terminase large subunit and **(C)** major capsid protein of the phage.

The whole-genome sequencing results showed that the whole-genome size of phage vB_KpnS_MK54 was 46,218 bp, and the G + C content was 48.29%. The results were uploaded to NCBI, and the accession number, MW119258, was obtained. Through Blastn online comparison, the results showed that the whole genome of MK54 is most similar to Klebsiella phage BUCT610 (GenBank no.: MZ318367.1). GeneMark S was used to predict protein-coding genes of phage MK54 genome, in which 32 CDSs were known protein functions and 48 CDSs were imaginary protein sequences (see Supplementary Table 1), and the CG View was used to visualise the prediction results (Figure 2A). Genes gp26 (holin) and gp80 (lysin) constitute the major lysis system of phage.

In this study, the conserved and evolutionary significance of the major capsid protein (gp16) and terminase large subunit (gp65) amino acid sequences were selected to construct a phylogenetic tree (Figures 2B,C; Luo et al., 2021). The figure shows that MK54 in the two phylogenetic trees is on the same branch as *K. pneumoniae* phage ZCKP8 and is closely related to phage BUCT610. Furthermore, ZCKP8 and BUCT610 belong to the unclassified subfamily of the Siphoviridae family, while MK54 is closely related to other phages. Therefore, we propose classifying it as a new subfamily.

### Plasmid Verification and Protein Expression

**Figure 3:**
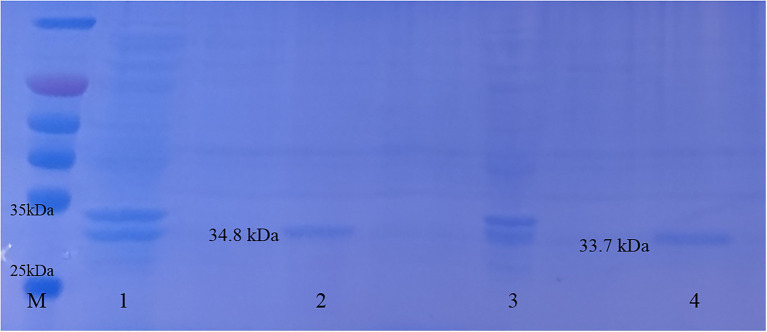
Endolysin LysG24 and LysCA protein expression. Purification results of LysG24 and LysCA recombinant protein. M, Protein molecular weight standard; 1, LysCA unpurified protein; 2, LysCA purified protein; 3, LysG24 unpurified protein; and 4, LysG24 purified protein.

The constructed plasmid was verified by PCR (see Supplementary Figure 1), and the results showed that LysG24 and LysCA were successfully ligated into the pET-32a(+) plasmid. SDS-PAGE analysis of the expressed protein (Figure 3) showed that LysG24 and LysCA were well expressed as soluble proteins in BL21. We overexpressed and purified endolysin, and the final concentration of the endolysin was 100 μg/ml.

### *In vitro* Antibacterial Activity

**Figure 4:**
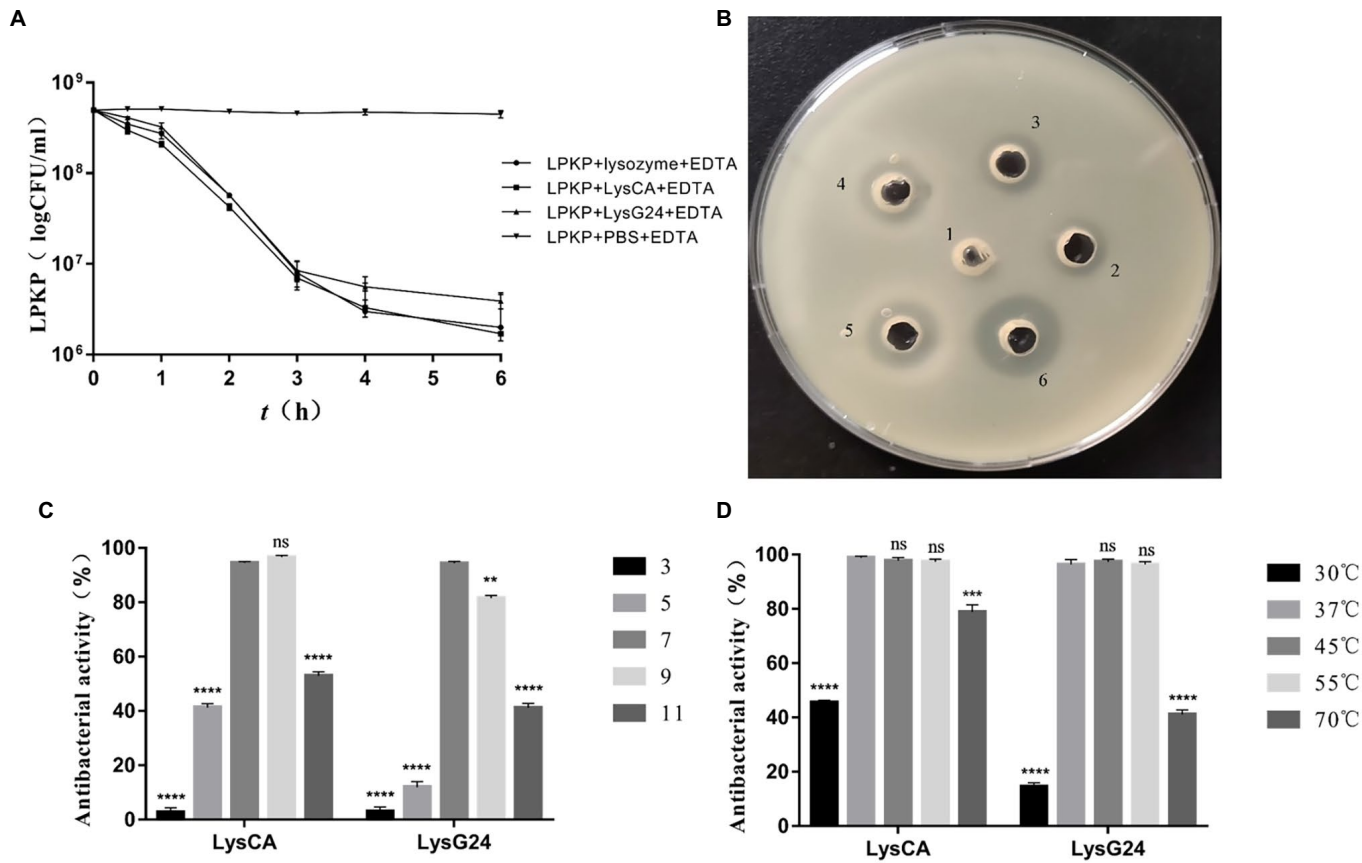
*In vitro* antibacterial effect of LysG24 and LysCA. **(A)** Antibacterial results of plate counting method. **(B)** Antibacterial results of plate diffusion method. 1, PBS + EDTA; 2, LysG24; 3, LysCA; 4, Lysozyme + EDTA; 5, LysG24 + EDTA; and 6, LysCA + EDTA. **(C)** Antibacterial activity of recombinant protein at different pH values. **(D)** Antibacterial activity of recombinant protein at different temperatures (^*^p < 0.05; ^**^p < 0.01; ^***^p < 0.005; ^****^p < 0.001; ns, no significant difference compared with the data at pH=7 or 37°C).

The results of the plate count test (Figure 4A) show that the concentration of LPKP can be reduced by 2.5 log values by LysCA and by 2.1 log values by LysG24, both of which have excellent antibacterial effects. The results of the plate diffusion test (Figure 4B) show that pure LysG24 (33.7 kDa) and LysCA (34.8 kDa) have a slight antibacterial effect on LPKP. However, adding EDTA as an outer membrane penetrant significantly improved the antibacterial effect. The antibacterial activity of LysG24 and LysCA at different temperatures (Figure 4C) and pH (Figure 4D) shows that the two endolysins also maintained high antibacterial activity in an alkaline environment. However, it is easy to lose endolysin activity in acidic environments. In addition, at 70°C, LysCA still had about 80% antibacterial activity and LysG24 had about 40% antibacterial activity, indicating that endolysins have high-temperature resistance. Meanwhile, the lysis profile test (Table 4) showed that the LysG24 and LysCA lysis profiles were the same: they can both lyse the indicator bacteria LPKP, as well as *E. coli* 1, SA 1 and SA 3. This indicates that LysG24 and LysCA have a broader lysis profile than vB_KpnS_MK54.

**Table 4:** Lysis profile of LysG24 and LysCA.

Number	Bacteria	Source	LysCA	LysG24
LPKP	*Klebsiella pneumoniae*	Forest musk deer	+	+
KP 1	*Klebsiella pneumoniae*	cattle	−	−
KP 2	*Klebsiella pneumoniae*	cattle	−	−
KP 3	*Klebsiella pneumoniae*	cattle	−	−
KP 4	*Klebsiella pneumoniae*	cattle	−	−
KP 5	*Klebsiella pneumoniae*	pig	−	−
PA 1	*Pseudomonas aeruginosa*	Forest musk deer	−	−
Bb 1	*Bordetella bronchiseptica*	Forest musk deer	−	−
KO 1	*Klebsiella oxytoca*	Forest musk deer	−	−
E.coli 1	*Escherichia coli*	Forest musk deer	+	+
E.coli 2	*Escherichia coli*	Forest musk deer	−	−
E.coli 3	*Escherichia coli*	cattle	−	−
SA 1	*Staphylococcus aureus*	Forest musk deer	+	+
SA 2	*Staphylococcus aureus*	Forest musk deer	−	−
SA 3	*Staphylococcus aureus*	cattle	+	+

+ Injection.

− Without injection.

### *In vivo* Antibacterial Activity

During the mouse challenge and treatment experiment, the clinical symptoms of the mice were observed every 2 h. After 48 h, all the experimental mice were anaesthetised and sacrificed, and necropsy results were observed. The results were as follows: the mice in group A (blank), group C (LysCA safety) and group D (LysG24 safety) were in a good mental state. Further, no death occurred during the experiment, and there were no abnormalities in lung tissue after autopsy. Mice in group B (bacterial) were infected with LPKP *via* the intranasal route. After 6 h of challenge, the mice began to show symptoms such as listlessness, slow movement, reduced feed intake and purulent secretions around their eyes. After 24 h of the challenge, two mice died, and after 48 h of the challenge, the other mice stopped performing actions and grouped together due to fear of cold. There was obvious consolidation of lung tissue with a small amount of bleeding after necropsy. The mice in group E (LysCA treatment) were administered intranasal LysCA injection at the onset of clinical symptoms, and the clinical symptoms were significantly relieved within 24 h. Within 48 h, the mice returned to normal, but their mental condition and mobility were still inferior to those in group A. Moreover, no abnormalities were found in the lung tissue after necropsy. The mice in group F (LysG24 treatment) received intranasal LysG24 injection when clinical symptoms appeared, and the clinical symptoms were partially relieved within 24 h. Within 48 h, the mental status and mobility of mice were recovered, but the recovery effect was not as good as that in group E. There was slight congestion and oedema in the lung tissue after necropsy.

**Figure 5:**
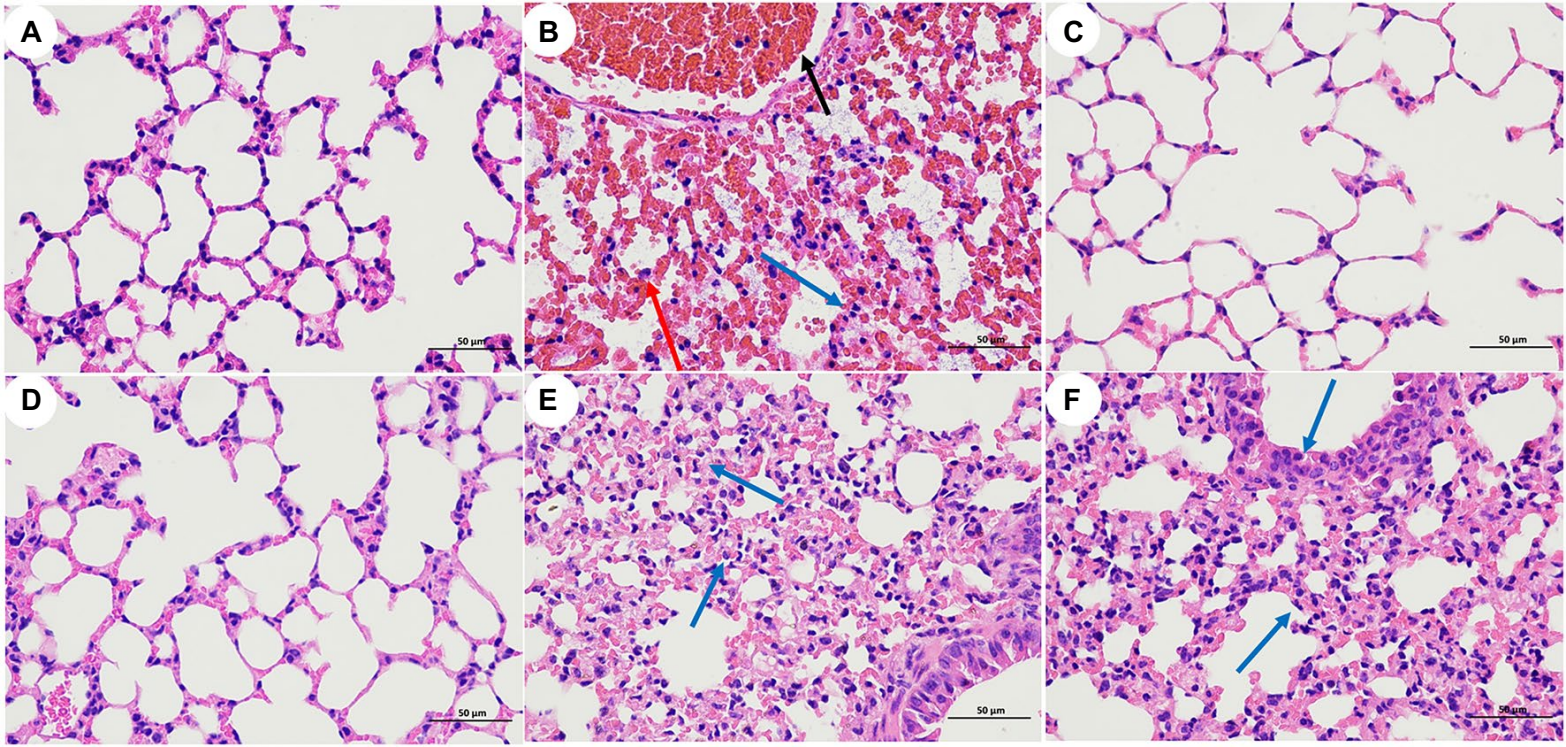
Pathological section of mouse lung tissue (H.E, 400×). **(A)** Blank control group. **(B)** Bacterial challenge group. **(C)** LysCA safety group. **(D)** LysG24 safety group. **(E)** LysCA treatment group. **(F)** LysG24 treatment group. Necrotic cell fragments (red arrows); Inflammatory cells (blue arrow); and Bleeding (black arrow).

Pathological sections of mouse lung tissue are shown in Figure 5. The results showed that the lung tissues of the mice in group A (blank), group C (LysCA safety) and group D (LysG24 safety) were normal with no lesions. The pathological results of group B (bacterial) mice showed large areas of tissue necrosis, congestion, haemorrhage and a large amount of inflammatory cell infiltration. The pathological results of group E (LysCA treatment) and group F (LysG24 treatment) mice showed mild thickening of the alveolar walls and a small amount of inflammatory cell infiltration. Combined with the lung lesion scores (Table 5), the pathological scores of the treatment group were lower than those of the bacterial group.

**Table 5:** Grading of histopathological features in the lungs of mice.

Group	Inflammatory	Bodiness	Congestion	Haemorrhage
A	0	0	0	0
B	4	0	4	3
C	0	0	0	0
D	0	0	0	0
E	1	1	0	0
F	1	1	0	0

No lesions or very few lesions were scored as 0; slight lesions were scored as 1; moderate lesions were scored as 2; severe lesions were scored as 3; and very severe lesions were scored as 4.

**Figure 6:**
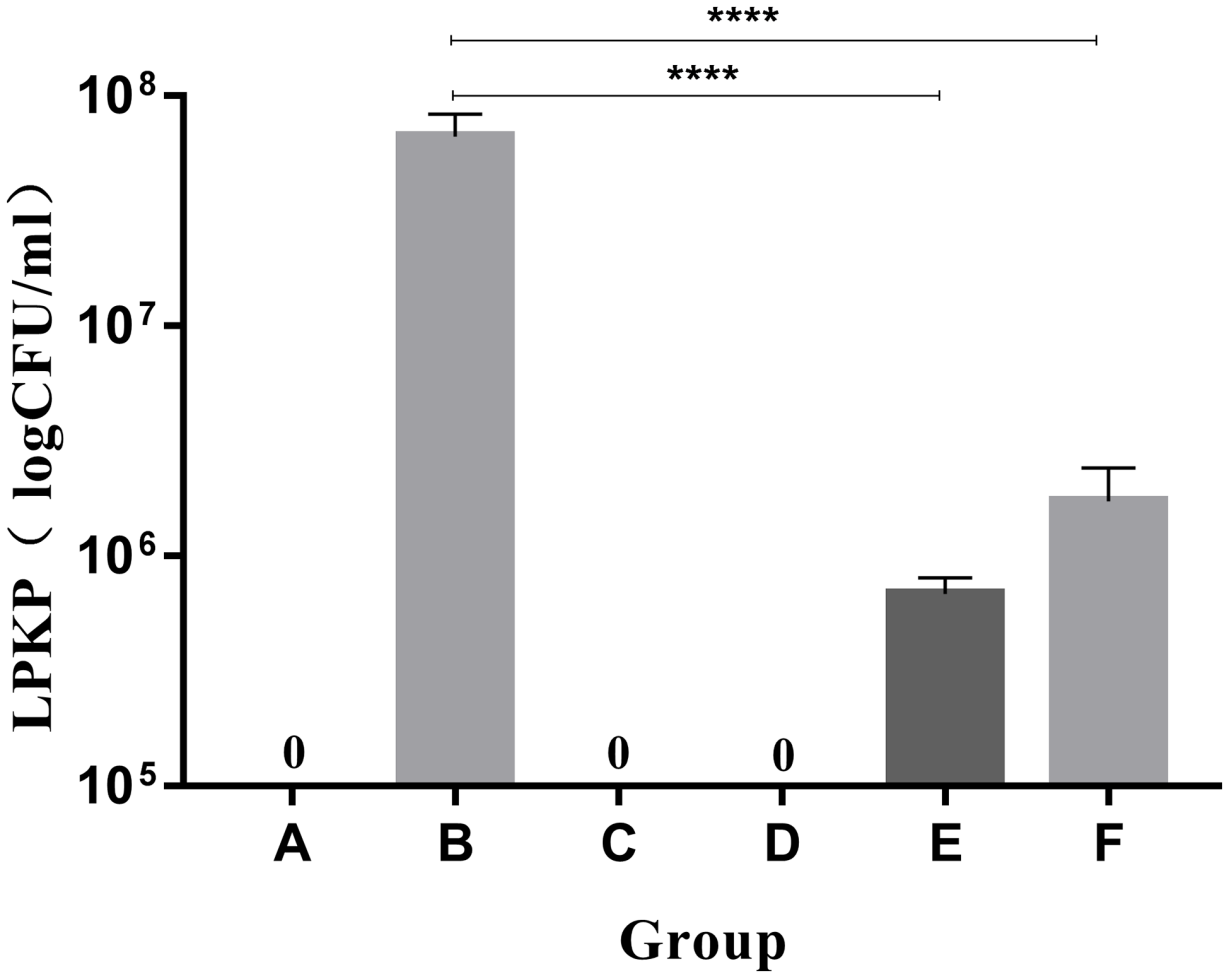
Bacterial load of mouse lung tissue (^*^p < 0.05; ^****^p < 0.001 compared with the bacteria group).

LPKP was not isolated from the lung tissues of mice in groups A (blank), C (LysCA safety) or D (LysG24 safety) according to pulmonary tissue bacterial load results (Figure 6). The concentration of LPKP isolated from group B was 6.67 × 10^7^ CFU/ml. The bacterial load of group E (LysCA treatment) was 6.87 × 10^5^ CFU/ml, which reduced the concentration of about 2 log of the host bacteria. The bacterial load of group F (LysG24 treatment) was 1.73 × 10^6^ CFU/ml, which reduced the host bacterial concentration by about 1.6 log. In conclusion, LysCA and LysG24 effectively reduced the proliferation of LPKP in mice, and LysCA had a significantly stronger antibacterial effect than LysG24 *in vivo*.

## Discussion

Bacteriophages are targeted bacterial viruses with multiple antibacterial effects, specifically invading and lysing host bacteria. Phage therapy has become a popular direction for bacterial therapy in the post-antibiotic era (Luong et al., 2020). In this study, LPKP was used as an indicator bacterium, and a lytic phage vB_KpnS_MK54 was isolated from the drinking water of an FMD farm (Evelien and Rodney, 2017). The polysaccharide depolymerase produced by the bacteriophage degrades the bacterial capsule to form a shallow halo around the plaque (Hughes et al., 1998). Biological characteristics tests showed that bacteriophage MK54 has good tolerance to temperature and pH, and there is a higher titre under strong alkali conditions. By measuring the MOI and one-step growth curve of the phage, the greatest benefit can be obtained by adjusting the amount of phage in future industrial processes (O’Flynn et al., 2004). The extremely low optimal MOI, short incubation period and large outbreaks indicate that MK54 has high proliferation efficiency and lytic activity. In addition, MK54 showed extremely high specificity in the lysis profile test. Therefore, bacteriophage MK54 has potential as a backup strain for bacteriophage therapy. When multiple phages attack the host bacteria at the same time, the process of bacteriophage lysis is inhibited, and the bacteria will become tolerant. Therefore, it is necessary to determine the biological characteristics of bacteriophages (Tomat et al., 2013; Yu et al., 2018; Zurabov and Zhilenkov, 2021). The *K. pneumoniae* phage MK54 has a double-stranded genome of 46,218 base pairs. The GC content of MK54 was 48.29%, which was significantly lower than the 57.32% of its host LPKP, which is similar to most bacteriophages. Compared with A and T/U, the synthesis of GC requires a higher energy cost. Therefore, phages that depend on the host for survival contain less GC than the host (Rocha and Danchin, 2002). Among the proteins with known functions, perforin (gp26) and endolysin (gp80) together constitute the major lysis system of phage, which cooperate to lyse the host bacteria. However, unlike most bacteriophages, the genes encoding lytic function proteins are not adjacent (Bai et al., 2013; Deng et al., 2021), which may also be the reason why the lysis period of MK54 is longer than that of other phages.

Bacteriophages have advantages in the treatment of bacterial diseases (Harada et al., 2018; Azam and Tanji, 2019), but there are also challenges. For example, rapid lysis of bacteria by phages may lead to the release of bacterial toxins. In the face of these challenges, some scholars have proposed that the use of phage lytic protein-endolysin instead of phage for bacterial treatment may help overcome these challenges (Doss et al., 2017). Endolysins can efficiently lyse bacteria without producing resistance. The endolysin molecular structure is known and the use of endolysin is safe and suitable for a wide range of applications (Parisien et al., 2008; Fischetti, 2018). Furthermore, endolysin has been widely confirmed to be effective in the lysis of Gram-positive bacteria (Yang et al., 2014; Vazquez et al., 2017). However, the outer membrane of Gram-negative bacteria prevents endolysin from approaching the peptidoglycan layer, making the ability of exogenously added endolysin to lyse bacteria very weak (Lim et al., 2014). Recently, natural endolysin was fused with the optimised N-terminal or C-terminal antimicrobial peptide to destroy the outer membrane, allowing the modified endolysin to have good antibacterial activity against Gram-negative bacteria (Briers et al., 2014b; Ma et al., 2017).

In this study, endolysin LysCA and LysG24 were successfully expressed, and their antimicrobial activity was detected *in vitro*. The results showed that endolysin alone had a slight ability to lyse LPKP. Notably, the antibacterial effect of endolysin was significantly improved by the addition of the outer membrane penetrant EDTA. In particular, the mixture of LysCA and EDTA exhibited the strongest lysis ability. It may be that the cecropin A residues and EDTA have a synergistic effect in destroying the outer membrane of Gram-negative bacteria, thereby enhancing the lysis of host bacteria by endolysin. Some studies have confirmed this synergistic effect, but the specific molecular mechanism remains to be studied (Yuksel et al., 2018; Lai et al., 2020; Huang et al., 2021). The optimal temperature and pH conditions for LysG24 and LysCA were investigated. Both LysG24 and LysCA exhibited high-temperature resistance and high activity in a wide range of pH environments. This is consistent with most reported high-temperature resistant lysins (Schmelcher et al., 2012; Sharma et al., 2016; Wu et al., 2018). Additionally, LysG24 and LysCA lysis profiles were similar. Thus, the modification of cecropin A residues only enhanced the lysis ability of endolysin but did not expand its lysis range. Compared with the lytic profile of the phage, the lytic profile of endolysin was wider. It had relatively more coverage for Gram-positive bacteria without outer membrane protection. In *in vitro* antibacterial tests, LysCA modified with cecropin A residues showed stronger bacterial lysis ability and environmental adaptability compared to that of natural endolysin LysG24. These results are similar to those of several studies on endolysins (Yan et al., 2019; Antonova et al., 2020). In addition, endolysin is highly dynamic in its natural state. The stability of the endolysin changes continuously with changes in pH (Sharma et al., 2016).

In recent years, endolysin therapy has gradually advanced from basic *in vitro* experiments to animal testing (Wang et al., 2017; Jasim et al., 2018; Gutierrez et al., 2020). The present study mainly evaluated the therapeutic effect of endolysin in a mouse model of pneumonia based on clinical observation and pathological analysis. LPKP was used to establish an experimental pneumonia model by instilling a bacterial dose *via* the intranasal route. According to the clinical symptoms and lung pathological section results, it was clear that the mice in the treatment group had significantly lower damage than those in the bacteria group. To more clearly show that LPKP is inhibited and lysed by endolysin in mice, we counted the bacterial load in the lungs of mice. The assay results showed that LysCA and LysG24 can effectively inhibit the growth of the host bacteria LPKP *in vivo* and relieve inflammation in the lung tissue, similar to the results of several previous *in vivo* studies of endolysin therapy (Peng et al., 2019; Dhungana et al., 2021). As expected, based on both the clinical signs and the bacterial load in the lungs of mice, LysCA showed a better therapeutic effect. This also shows that by combining different peptides with endolysin, an antibacterial lysin with the desired properties and robust activity can be obtained (Oliveira et al., 2018; Murray et al., 2021). In addition, the safety test results showed that LysG24 and LysCA endolysin had no clinical toxicity in mice.

However, LysG24 and LysCA have a drawback in that they require the assistance of EDTA to effectively invade and destroy host bacteria. The role of EDTA, an acidic chelating agent, accumulating in the organism is unclear. In addition, the animal test in this study was only a preliminary study, which may differ greatly from clinical practice results.

Taken together, LysG24 and LysCA showed excellent antimicrobial activity *in vivo* and *in vitro*, both of which have potential therapeutic effects against LPKP-induced infections. Moreover, LysG24 and LysCA were sufficiently safe for use in BALB/C mice. In particular, LysCA modified with cecropin A residues exhibited stronger antibacterial capacity and better environmental adaptation than LysG24. In short, engineered endolysins are excellent candidates for new antibacterial drugs in the future.

## Conclusion

In conclusion, we report the main biological characteristics and genome-wide analysis of the isolated *K. pneumoniae* phage vB_KpnS_MK54. Genetic evolution analysis showed that the phage belongs to the family Siphoviridae. Whole-gene sequencing was also conducted to determine phage protein content. We reported the natural endolysin LysG24 and a newly engineered LysCA endolysin. They showed strong antibacterial activity against *K. pneumoniae* both *in vivo* and *in vitro*. In addition, the endolysins had no toxic effects on the animal body, indicating that endolysin therapy can become a future new type of treatment for bacterial infections.

## Data Availability Statement

The datasets presented in this study can be found in online repositories. The names of the repository/repositories and accession number(s) can be found at: https://www.ncbi.nlm.nih.gov/genbank/, MW119258.1.

## Ethics Statement

The animal study was reviewed and approved by the Animal Protection Law of the People’s Republic of China and approved by the National Institute of Animal Health Animal Care and Use Committee at Sichuan Agricultural University (No. SYXK2019-187).

## Author Contributions

BL and XY conceived the project, designed the experiments, analysed the data, and contributed equally to this study. GH, ZL, and JZ performed the majority of the experiments. BL wrote the manuscript. SC, YW, YL, ZY, and MR supervised the work and edited the final version of the manuscript. All authors have read and approved the final manuscript.

## Funding

This work was funded by the double branch plan of the discipline construction of Sichuan Agricultural University (3572070), the Sichuan Department of Science and Technology Support Project (2019YJ0650) and the innovation research group on quality and safety of featured agricultural products in Three Gorges Reservoir Area (CXQTP19037).

## Conflict of Interest

The authors declare that the research was conducted in the absence of any commercial or financial relationships that could be construed as a potential conflict of interest.

## Publisher’s Note

All claims expressed in this article are solely those of the authors and do not necessarily represent those of their affiliated organizations, or those of the publisher, the editors and the reviewers. Any product that may be evaluated in this article, or claim that may be made by its manufacturer, is not guaranteed or endorsed by the publisher.

## Footnotes

^1^ http://topaz.gatech.edu/GeneMark/

^2^ http://github.com/bbuchfink/diamond

^3^ http://cgview.ca/
