# Supplementary material for: Isolation of Klebsiella pneumoniae Phage vB_KpnS_MK54 and Pathological Assessment of Endolysin in the Treatment of Pneumonia Mice Model
Source: Front Microbiol. 2022 Mar 21;13:854908. doi: 10.3389/fmicb.2022.854908 (PMC8978833; doi:10.3389/fmicb.2022.854908)
Supplement: Supplementary file 1 [file Data_Sheet_1.docx]

Supplementary Table 1 Genes with proposed functions in the genome of phage vB_KpnS_MK54

| Gene | Feature | Nucleotide position | | Length(bp) | Strand | Function |
| --- | --- | --- | --- | --- | --- | --- |
|  |  | Start | End |  |  |  |
| gp1 | CDS | 128 | 622 | 495 | + | tail protein |
| gp2 | CDS | 804 | 1016 | 213 | - | hypothetical protein |
| gp3 | CDS | 1016 | 1378 | 363 | - | hypothetical protein |
| gp4 | CDS | 1378 | 1572 | 195 | - | hypothetical protein |
| gp5 | CDS | 1572 | 1958 | 387 | - | hypothetical protein |
| gp6 | CDS | 1961 | 2155 | 195 | - | hypothetical protein |
| gp7 | CDS | 2155 | 2355 | 201 | - | hypothetical protein |
| gp8 | CDS | 2452 | 2667 | 216 | + | hypothetical protein |
| gp9 | CDS | 2751 | 2942 | 192 | + | tumor suppressor protein |
| gp10 | CDS | 3063 | 3674 | 612 | + | endonuclease |
| gp11 | CDS | 3724 | 4134 | 411 | + | hypothetical protein |
| gp12 | CDS | 4200 | 4397 | 198 | + | hypothetical protein |
| gp13 | CDS | 4394 | 4738 | 345 | + | hypothetical protein |
| gp14 | CDS | 4921 | 6321 | 1401 | + | Prohead core protein serine protease |
| gp15 | CDS | 6321 | 6785 | 465 | + | capsid stabilizing protein |
| gp16 | CDS | 6797 | 7885 | 1089 | + | major capsid protein |
| gp17 | CDS | 7928 | 8308 | 381 | + | hypothetical protein |
| gp18 | CDS | 8537 | 9355 | 819 | + | hypothetical protein |
| gp19 | CDS | 9385 | 9636 | 252 | - | hypothetical protein |
| gp20 | CDS | 9662 | 9835 | 174 | - | hypothetical protein |
| gp21 | CDS | 9835 | 9972 | 138 | - | hypothetical protein |
| gp22 | CDS | 9972 | 10787 | 816 | - | putative transcriptional regulator |
| gp23 | CDS | 10795 | 10911 | 117 | - | hypothetical protein |
| gp24 | CDS | 10908 | 11249 | 342 | - | hypothetical protein |
| gp25 | CDS | 11323 | 11481 | 159 | - | hypothetical protein |
| gp26 | CDS | 11480 | 11767 | 288 | + | holin |
| gp27 | CDS | 11781 | 12137 | 357 | + | head to tail adaptor |
| gp28 | CDS | 12256 | 12600 | 345 | + | Minor capsid protein |
| gp29 | CDS | 12616 | 13020 | 405 | + | Minor capsid protein |
| gp30 | CDS | 13017 | 13400 | 384 | + | tail completion protein |
| gp31 | CDS | 14016 | 14771 | 756 | + | major tail subunit |
| gp32 | CDS | 14793 | 15014 | 222 | - | hypothetical protein |
| gp33 | CDS | 15153 | 15545 | 393 | - | NinX protein |
| gp34 | CDS | 15542 | 15676 | 135 | - | hypothetical protein |
| gp35 | CDS | 15774 | 16481 | 708 | + | hypothetical protein |
| gp36 | CDS | 16481 | 19423 | 2943 | + | putative tail protein |
| gp37 | CDS | 19423 | 19893 | 471 | + | minor tail protein |
| gp38 | CDS | 19893 | 20363 | 471 | + | hypothetical protein |
| gp39 | CDS | 20489 | 20719 | 231 | + | hypothetical protein |
| gp40 | CDS | 20756 | 23212 | 2457 | + | tail protein |
| gp41 | CDS | 23251 | 25335 | 2085 | + | tail fiber protein |
| gp42 | CDS | 25364 | 25831 | 468 | - | single-stranded DNA-binding protein |
| gp43 | CDS | 25841 | 26878 | 1038 | - | P-loop containing nucleoside triphosphate hydrolase |
| gp44 | CDS | 26908 | 27885 | 978 | - | exonuclease |
| gp45 | CDS | 27882 | 28139 | 258 | - | tail fibers protein |
| gp46 | CDS | 28172 | 28519 | 348 | - | Nuclease |
| gp47 | CDS | 28584 | 30500 | 1917 | - | DNA helicase |
| gp48 | CDS | 30593 | 31486 | 894 | + | DNA primase |
| gp49 | CDS | 31501 | 31797 | 297 | - | hypothetical protein |
| gp50 | CDS | 31797 | 33350 | 1554 | - | hypothetical protein |
| gp51 | CDS | 33350 | 33583 | 234 | - | hypothetical protein |
| gp52 | CDS | 33808 | 33987 | 180 | + | hypothetical protein |
| gp53 | CDS | 34027 | 34140 | 114 | + | hypothetical protein |
| gp54 | CDS | 34180 | 34302 | 123 | + | hypothetical protein |
| gp55 | CDS | 34316 | 34501 | 186 | + | hypothetical protein |
| gp56 | CDS | 34517 | 34795 | 279 | + | hypothetical protein |
| gp57 | CDS | 34802 | 35236 | 435 | + | hypothetical protein |
| gp58 | CDS | 35340 | 35495 | 156 | + | hypothetical protein |
| gp59 | CDS | 35495 | 35785 | 291 | + | hypothetical protein |
| gp60 | CDS | 35782 | 36495 | 714 | + | hypothetical protein |
| gp61 | CDS | 36492 | 36584 | 93 | + | hypothetical protein |
| gp62 | CDS | 36588 | 36746 | 159 | + | hypothetical protein |
| gp63 | CDS | 36873 | 37388 | 516 | + | homing endonuclease |
| gp64 | CDS | 37470 | 37949 | 480 | + | hypothetical protein |
| gp65 | CDS | 38087 | 39511 | 1425 | + | large terminase subunit |
| gp66 | CDS | 39532 | 39822 | 291 | - | hypothetical protein |
| gp67 | CDS | 39911 | 40150 | 240 | + | hypothetical protein |
| gp68 | CDS | 40147 | 40329 | 183 | + | replicase polyprotein |
| gp69 | CDS | 40329 | 40625 | 297 | + | hypothetical protein |
| gp70 | CDS | 40622 | 40810 | 189 | + | hypothetical protein |
| gp71 | CDS | 40810 | 41010 | 201 | + | hypothetical protein |
| gp72 | CDS | 41020 | 41178 | 159 | + | hypothetical protein |
| gp73 | CDS | 41244 | 42680 | 1437 | + | portal protein |
| gp74 | CDS | 42709 | 43608 | 900 | + | Minor capsid protein |
| gp75 | CDS | 43620 | 43748 | 129 | - | hypothetical protein |
| gp76 | CDS | 43853 | 44734 | 882 | - | hypothetical protein |
| gp77 | CDS | 44731 | 44862 | 132 | - | hypothetical protein |
| gp78 | CDS | 44862 | 45152 | 291 | - | hypothetical protein |
| gp79 | CDS | 45207 | 45590 | 384 | - | hypothetical protein |
| gp80 | CDS | 45692 | 46129 | 438 | + | lysozyme |

Supplementary Figure 1 Endolysin LysG24 and LysCA plasmid construction


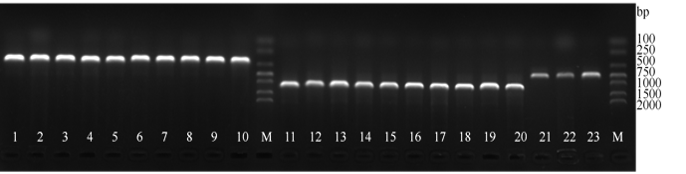


Construction of LysG24 and LysCA recombinant plasmids. M, DL2000 DNA Marker; 1-5, LysCA primer (481 bp); 6-10, LysG24 primer (456 bp); 11-15, LysG24 recombinant plasmid (1168 bp); 16-20, LysCA recombinant plasmid (1193 bp); 21-23, pET-32a (+) empty plasmid (712 bp).
